# Immune System in the Brain: A Modulatory Role on Dendritic Spine Morphophysiology?

**DOI:** 10.1155/2012/348642

**Published:** 2012-04-03

**Authors:** Oscar Kurt Bitzer-Quintero, Ignacio González-Burgos

**Affiliations:** ^1^Laboratorio de Neuroinmunomodulación, División de Neurociencias, Centro de Investigación Biomédica de Occidente, Instituto Mexicano del Seguro Social, Sierra Mojada No. 800 Col. Independencia, 44340 Guadalajara, JAL, Mexico; ^2^Laboratorio de Psicobiología, División de Neurociencias, Centro de Investigación Biomédica de Occidente, Instituto Mexicano del Seguro Social, Sierra Mojada No. 800 Col. Independencia, 44340 Guadalajara, JAL, Mexico; ^3^Departamento de Biología Celular y Molecular, CUCBA, Universidad de Guadalajara, 45110 Zapopan, JAL, Mexico

## Abstract

The central nervous system is closely linked to the immune system at several levels. The brain parenchyma is separated from the periphery by the blood brain barrier, which under normal conditions prevents the entry of mediators such as activated leukocytes, antibodies, complement factors, and cytokines. The myeloid cell lineage plays a crucial role in the development of immune responses at the central level, and it comprises two main subtypes: (1) resident microglia, distributed throughout the brain parenchyma; (2) perivascular macrophages located in the brain capillaries of the basal lamina and the choroid plexus. In addition, astrocytes, oligodendrocytes, endothelial cells, and, to a lesser extent, neurons are implicated in the immune response in the central nervous system. By modulating synaptogenesis, microglia are most specifically involved in restoring neuronal connectivity following injury. These cells release immune mediators, such as cytokines, that modulate synaptic transmission and that alter the morphology of dendritic spines during the inflammatory process following injury. Thus, the expression and release of immune mediators in the brain parenchyma are closely linked to plastic morphophysiological changes in neuronal dendritic spines. Based on these observations, it has been proposed that these immune mediators are also implicated in learning and memory processes.

## 1. Introduction

Cell survival in the central nervous system (CNS) has been closely associated with activation of the immune system, a tightly controlled process [1]. The CNS parenchyma is separated from the bloodstream by the blood brain barrier (BBB), the integrity of which is maintained by endothelial tight junctions. Under normal conditions, the BBB prevents the entry into the brain parenchyma of leukocytes, antibodies, complement factors, and cytokines, as well as antigen presenting cells (APCs) such as dendritic cells, B cells, and macrophages [1, 2].

**Figure 1:**
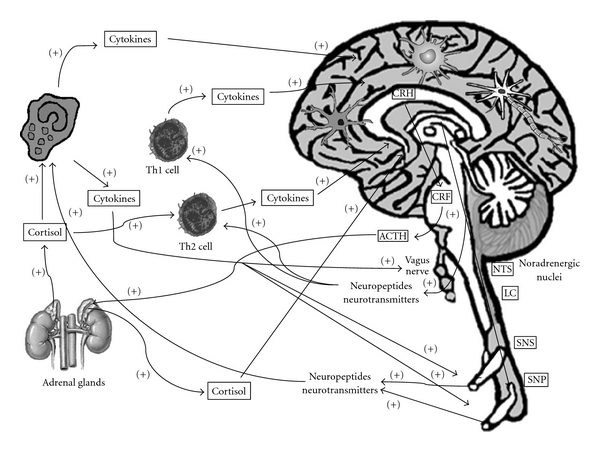
A bidirectional relationship links the immune and nervous systems, which communicate through the synthesis and the release of mediators such as cytokines, prostanoids, neuropeptides, and neurotransmitters. CRH: corticotropin-releasing hormone. CRF: corticotropin-releasing factor. ACTH: adrenocorticotropic hormone. NTS: nuclei of the solitary tract. LC: locus coeruleus. SNS: sympathetic nervous system. PNS: parasympathetic nervous system.

In the CNS, the immune system mediates several anatomical and physiological adaptations to protect its vital functions from the damage caused by inflammation [3] (Figure 1). In addition to the BBB and the cerebrospinal fluid (CSF), which also prevents the entry of plasma protein and immune cells into the parenchyma [3, 4], other mechanisms appear to sustain the “immune privilege” of the CNS, including local production of anti-inflammatory molecules such as transforming growth factor (TGF-*β*), melanocyte-stimulating hormone-alpha (*α*-MSH), and vasoactive intestinal peptide (VIP), as well as the constitutive expression of the Fas ligand (FasL or CD95L) that mediates the infiltrating immune cells death in the CNS [3, 4].

Studies of CNS damage have predominantly focused on neurons, as these cells determine the survival and function of the CNS [5–7]. However, in recent decades, additional cell subtypes have been seen to be involved in maintaining CNS homeostasis [7]. Microglial cells can generate an immune response after damage and alter nerve cell and dendritic spine morphophysiology. Similarly, dendritic spines may play a role in such events, these spines specializing in translating afferent excitatory synaptic inputs to postsynaptic neurons. Plastic changes in these structures are closely associated with the processing of cognitive-related afferent information. Accordingly, alterations in dendritic spine morphophysiology induced by microglia-associated immune responses exert deleterious effects on the transmission of mnemonic information, although the underlying mechanisms remain poorly understood. 

## 2. Immune Cells in the Nervous System

**Figure 2:**
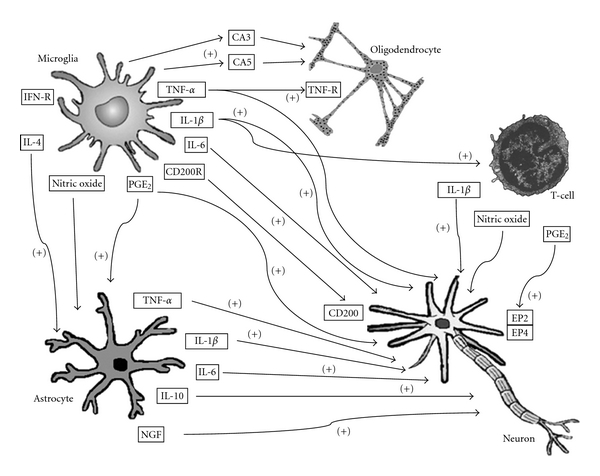
Microglia are activated in the brain parenchyma and mediate the synthesis and release of proinflammatory and anti-inflammatory cytokines, prostanoids, complement system molecules, and free radicals such as nitric oxide, affecting other cells including astrocytes, oligodendrocytes, and neurons. The signal established between microglial cells and neurons through the expression of CD200-CD200R maintains the microglia in a nonactivated state, and, when this signal is interrupted, microglia are immediately activated and initiate the generation and release of inflammatory mediators. PGE2: prostaglandin-E2; IFN-R: interferon-receptor; TNF-*α*: tumor necrosis factor-alpha; TNFR: tumor necrosis factor receptor; IL-1*β*: interleukin-1 beta; IL-6: interleukin-6; IL-10: interleukin-10; IL-4: interleukin-4; NGF: nerve growth factor.

The monocyte/macrophage cell lineage plays a key role in the development of innate and adaptive immune responses. Furthermore, some cell subpopulations associated with this lineage exhibit highly specialized phagocytic activity following cell degradation [8]. Two major subtypes of CD45^+^ cells have been described in the myeloid lineage: the microglia resident population that is distributed throughout the brain parenchyma; perivascular macrophages located in the brain capillaries of the basal lamina and the choroid plexus. Parenchymal microglia cells are precursor cells derived from CD45^+^ bone marrow cells that migrate into the CNS in the early stages of development, and they are capable of self-renewal within the brain parenchyma [7, 8]. By contrast, perivascular macrophages replace bone marrow elements at a more rapid rate and they have more features of monocytes/macrophages than parenchymal microglia [7, 8]. To date, no specific markers for these cell types have been described, although perivascular macrophages express higher levels of CD45, major histocompatibility complex (MHC-II), costimulatory molecules, and receptor recognition patterns than CD14 cells. The levels of these mediators are slightly lower in parenchymal microglial cells [8] (Figure 2).

Glial cells are the main source of many of the resident cytokines in the CNS. In addition to being “target” cells for cytokines, glial cells release important amounts of neuroactive substances that promote neuronal survival, such as neurotrophins and growth factors. By contrast, microglia release potentially neurotoxic substances, including nitric oxide (NO), reactive oxygen species (ROS), proteases, excitatory amino acids, and cytokines [9].

Activated macrophages (MQs) and microglia represent a major source of proinflammatory cytokines and chemokines within the CNS. These cells release reactive nitrogen species (RNS) and ROS and produce several factors that exert cytotoxic effects on oligodendrocytes and neurons [7]. Activated microglia can provoke excess MHC-II expression, facilitating its involvement in events mediated by the immune system. Microglial activation strongly influences the profile of cytokines released by these cells through two distinct mechanisms: pattern recognition receptors (PRRs; molecular traces remaining after a pathogenic insult) and immune response activation [6]. Activation of the monocyte/macrophage cell lineage through PRRs represents an important component of the innate immune response, which induces the production of proinflammatory cytokines and chemokines like IL-8, monocyte chemoattractant protein-1 (MCP-1), macrophage inflammatory protein-1 (MIP-1*α*), and MIP-1*β*. Moreover, other forms of PRR can activate regulatory cytokines within the CNS, such as interleukin-4 (IL-4) and interleukin-10 (IL-10) [6, 7].

Microglia located in perivascular areas can act as antigen presenting cells (APCs.) These cells evolve from their resting state and release significant quantities of Th1 cytokines, such as tumor necrosis factor-alpha (TNF-*α*) and IL-12 [7]. Such perivascular microglia alerts the parenchymal microglia to the signals of “damage,” indicating the presence of inflammatory cells in the perivascular space. This provokes the activation of the parenchymal microglia, which proliferate and produce effector cells that eliminate these damage signals and that restore the tissue integrity [7]. Microglia can exhibit diverse activation states, including migratory, phagocytic, and regenerative. These different functional states are regulated by positive and negative feedback loops and by microenvironment damage signals [7]. Activated microglia in turn activate several proinflammatory cytokines, anti-inflammatory cytokines (IL-10, TGF-*β*, IL-1Ra), prostanoids (prostaglandin-E_2_ or PGE_2_, prostaglandin-D_2_ or PGD_2_ and thromboxane-A_2_ or TXA_2_), cytotoxic molecules (ROS, RNS, inducible nitric oxide synthase or iNOS), and chemokines (IL-8, IL-10, MCP-1, MIP-1*α*, MIP-1*β*, MIP-3*β*, MDC, RANTES) [5, 10, 11].

The contribution of astrocytes to the neuroimmune system is even more complex than that of microglia. This population plays an essential role in neuronal life-support, and it is essentially divided into 2 main subtypes: fibrous astrocytes located in the white matter; protoplasmic astrocytes located in the gray matter, and they contribute to the BBB [9, 12, 13]. Some of the most important functions of astrocytes are related to the regulation of ion transport and neurotransmitter concentrations and the transmission of electric impulses. Astrocytes also act as neuroprotectors by secreting neurotrophins and releasing potentially toxic inflammatory molecules [9] (Figure 2). The third type of glial cells is the oligodendrocyte, which participates actively in myelination and is also involved in the secretion of specific inflammatory molecules [7] (Figure 2).

The microvasculature is a key element in cerebral damage. Endothelial cells represent an important source of immune mediators such as prostanoids and nitric oxide, both of which are implicated in the immune cell adhesion process [9]. Damage to the BBB can result in increased permeability, facilitating leukocyte access to the cerebral parenchyma, inducing the release of neurotoxins and endogenous inflammatory mediators and promoting phagocytosis of cell debris by perivascular MQs [9].

Diseases of the nervous system result in the upregulation of MHC-II and adhesion-costimulation molecules (CD11a, CD40, CD54, CD80, CD86) in activated microglia, indicating that these cells may acquire APC activity and actively participate in T-cell restimulation [14, 15]. This suggests the existence of a complex interaction network linking microglia, astrocytes, and invader T cells and involving a balance between Th1/Th2 signals, which defines the immune response of the CNS [14, 16].

*In vivo* microglial activation is regulated by several neuron-derived molecules, such as the CD200 marker, whilst microglia expresses CD200R. When CD200 expression is suppressed as a result of cell damage or after pathogenic stimulus, microglia switch to an activated phenotype, inducing the expression of the CD45 receptor, MHC-II, and the complement receptor 3 [17]. Resident microglia perform a wide variety of highly specialized functions in the cerebral parenchyma. These include the modulation of synaptic strength and efficacy through the remodeling of synaptic architecture [10] and induction of new synapses through regulation of synaptogenesis at the early stages of brain development. Microglia have also been suggested to regulate secretion of thrombospondin by astrocytes and secretion of the T-cell-derived serine protease (TSP-1). TSP-1 interacts with integrins that are associated with the CD47 protein and with the regulatory protein SRIPa, a transmembrane protein expressed in neurons and macrophages. The SRIPa-CD47 system regulates migration, phagocytosis, immune homeostasis, and the maintenance of the neuronal network [10].

Microglia play a key role in restoring neuronal connectivity following damage, by controlling reactive synaptogenesis [10]. Microglial cells may influence the homeostatic process of “synaptic scaling” that provides the adjustment in the strength of all synapses through distinct mechanisms in response to long-lasting changes in neuronal activity [10]. In addition, microglia are intimately involved in the development of the nervous system and in controlling the balance between neurogenesis and neuronal death. As mentioned previously, these cells play a dual role being implicated both in neurogenesis and neuronal death. While the mechanisms underlying this dichotomy remain unclear, differences in the activation state of microglia [10, 18] and the differential release of cytotoxic or cyto protective factors [18] have been reported. Thus, we conclude that microglia have the potential to regulate the development and function of neuronal networks by constantly monitoring the “status” of synaptic contacts and integrating new information [10].

## 3. The Relationship between the Immune System and Dendritic Spines

The inflammatory process observed in the CNS during either sepsis or endotoxic shock involves a wide variety of molecules that regulate the synthesis and release of inflammatory mediators, such as proinflammatory cytokines, prostanoids, and cytotoxic molecules. Such events are triggered both in the peripheral and the central nervous systems. These mediators are also generated and released during excitotoxicity induced by excessive glutamate release, ischemia reperfusion, brain trauma and neurodegenerative processes such as those associated with multiple sclerosis, Alzheimer's and Parkinson's diseases, leading to the activation of common inflammatory pathways [15–17, 19]. One such mediator is TNF-*α*, which is released by nerve cells and modifies the firing rate and excitability of neurons, thereby affecting processes such as long-term potentiation (LTP) and long-term depression (LTD) [15–17, 19].

**Figure 3:**
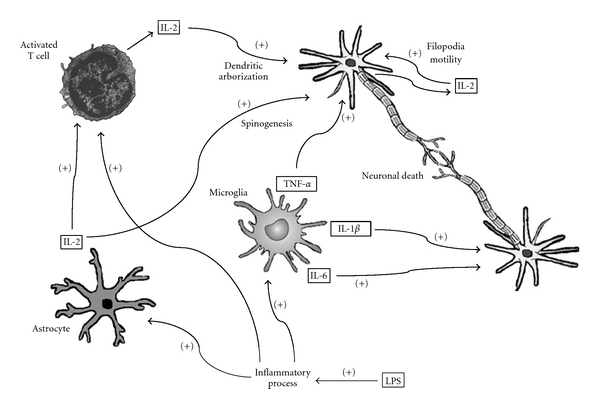
Interleukin-2 is involved in developmental processes such as neuronal dendritic arborization, and it induces a significant increase in spinogenesis and dendritic filopodia motility. Activated microglia release significant amounts of inflammatory mediators that induce neuronal death, synapse remodeling, and destabilization of synaptic contacts. IL-2: interleukin-2; TNF-*α*: tumor necrosis factor-alpha; IL-1*β*: interleukin-1 beta; IL-6: interleukin-6; LPS: lipopolysaccharide.

The complex neural networks in the mammalian forebrain are regulated by interactions with glia cells (Figure 3), but the underlying mechanisms are poorly understood. Microglia may induce synaptic remodeling though direct contact or via the release of soluble factors (cytokines) that destabilize nearby synapses [19]. Microglia are thought to directly modulate dendritic spine dynamics in response to lipopolysaccharide (LPS) administration [19], which in mice results in a significant long-term upregulation of the expression of genes involved in the release and metabolism of arachidonic acid (AA) in the hippocampus [20, 21]. AA is a precursor of prostaglandins, thromboxanes, and leukotrienes, biochemical mediators of the inflammatory process responsible for endotoxic shock [20]. At a dose of 1 mg/kg body weight, intraperitoneal administered LPS to rats and mice induces “sickness behavior,” changes in temperature and oxidative stress in the brain [20, 22, 23]. Likewise, acute peripheral administration of LPS is sufficient to trigger microglial activation, inducing the release of proinflammatory cytokines at both the peripheral and central levels [19, 24].

A recent study reported altered cognitive processes in a group of patients recovering from sepsis and endotoxic shock, including deficits in memory and attention [19]. LPS interacts with toll-like receptors (TLRs) at the MQs level, triggering the generation and release of a variety of important proinflammatory cytokines, including TNF-*α*, IL-1*β*, and IL-6. These proteins can cross the BBB to activate both microglia and astrocytes near brain capillaries. In addition, TNF-*α*, IL-1*β*, and IL-6 promote information flow via the vagus nerve, through the so-called inflammatory reflex. These responses promote the synthesis of PGs and cytokines in the brain parenchyma, which induce a fever state and sickness behavior [19]. Peripheral administration of LPS in rodents can affect certain cognitive functions by impairing LTP, a key physiological process in learning and spatial memory. LPS administration also induces peripheral inflammation, affecting the function of neural networks and glial cells. In addition, resident microglial cells in the brain parenchyma are activated, altering their phenotype and in turn affecting synaptic function [19].

One week after LPS administration, the dynamics of dendritic spines are comparable to those of control animals, indicating a minimal effect of acute systemic LPS treatment, at least in the short term. However, by 4 weeks after LPS administration, dendritic spine density increases to twice that of control animals. Moreover, this increase in spine density is associated with microglial activity, suggesting long-term effects on the CNS triggered by a transient peripheral immune response [19].

Acute induction of sickness behavior, which is accompanied by adverse effects on both emotional and cognitive processes, is at least partially responsible for the altered morphophysiological dynamics of dendritic spines [19, 24]. Cytokines released from glial cells can affect synaptic function, leading to alterations in spine morphology. Microglial cell proliferation has been reported in animal models of neurodegenerative disorders, accompanied by increased levels of proinflammatory cytokines and alterations in dendritic spine morphology and density [19]. Recent studies also suggest that the creation of an “inflammatory environment” may influence the generation of new synaptic connections. Indeed, microglia activated by inflammation may release cytokines and growth factors that “modulate” synaptic transmission, possibly by altering dendritic spine morphology. The inflammatory process also regulates functional synaptic connectivity, generating new connections in the adult brain [24].

Interestingly, new hippocampal neurons grown under conditions of chronic inflammation are more responsive to inhibitory afferent signals than those grown in normal conditions prior to the onset of inflammation. In the latter case, inflammation has no effect on the cell migration or polarity, on dendritic arborization or development, or on spine density, although dendritic spines in the new granule cells of the dentate gyrus exhibit very high mobility [24–26].

IL-2 is an immunoregulatory cytokine released from neurons and astrocytes in the CNS upon T-cell activation. This cytokine binds to receptors that are widely distributed throughout the brain. Following its release, IL-2 is thought to exert neuroregulatory effects and is implicated in various CNS disorders. In the hippocampus of experimental animals, IL-2 influences several cognitive processes, such as LTP, modifying the molecular substrates underlying learning and memory. In addition, IL-2 provides trophic support to rat hippocampal neurons in primary culture, with positive effects on morphology and neurite branching [25]. In cultured hippocampal neurons, IL-2 also affects dendritic development and spinogenesis by day 14 *in vitro*, increasing dendritic arborization and neuronal growth. These effects are more pronounced on day 7, when the cytokine significantly augments the motility of dendritic filopodia. Together, these findings suggest that IL-2 exerts promoter-like effects on dendritic development and spinogenesis in cultured hippocampal neurons at early developmental stages [25].

The hippocampal region is a very plastic structure, displaying fluctuations in dendritic arborization, as well as changes in dendritic spine density and neuronal cell body size. These and other plastic modifications of neuronal cytoarchitecture are heavily implicated in hippocampal-dependent learning and memory processes. Many regulatory and stress factors can affect neuronal circuitry and interfere with the development of dendritic trees. In this context, IL-2 increases the number and length of dendritic branches in neuronal primary culture on days 7, 10, and 14 *in vitro* [25]. The length, diameter, and arborization of dendritic trees significantly influence neuronal function, and, hence, by increasing dendritic arborization and length, the signal transmission between neurons and the function of local neuronal networks are modulated. Accordingly, such alterations are associated with the development of behavioral disorders [25].

The formation of filopodia is a highly dynamic process that occurs early in CNS development in mammals. These structures may be involved in the formation of new dendritic spines and synapses at later stages of development. The ability of IL-2 to increase filopodia motility is likely involved in the development of new dendrites [25, 27].

## 4. Conclusion

In conclusion, we propose a close relationship between cytokine expression and the regulation of dendritic spine dynamics within a microuniverse in which the immune system and nervous system are closely linked. These internal interdependent systems mediate nonspecific behavioral responses, as well as learning and memory processes. Further studies are required to fully determine the full nature and physiological outcome of the relationship between immune system activity and dendritic spine dynamics, as well as the potential implications for learning and memory processes.

## Abbreviations

*α*-MSH: Melanocyte stimulating hormone-alpha
AA: Arachidonic acid
APC: Antigen presenting cells
BBB: Blood brain barrier
CD: Cluster of differentiation
CNS: Central nervous system
CSF: Cerebrospinal fluid
iNOS: Inducible nitric oxide synthase
IL-1*β*: Interleukin-1 beta
IL-2: Interleukin-2
IL-4: Interleukin-4
IL-6: Interleukin-6
IL-10: Interleukin-10
LPS: Lipopolysaccharide
LTD: Long-term depression
LTP: Long-term potentiation
MQs: Macrophages
MHC: Major histocompatibility complex
MCP-1: Monocyte chemoattractant protein-1
MIP-1: Macrophage inflammatory protein-1
NO: Nitric oxide
PGs: Prostaglandins
PRRs: Pattern recognition receptors
RNS: Reactive nitrogen species
ROS: Reactive oxygen species
TGF*β*: Transforming growth factor
TNF-*α*: Tumor necrosis factor-alpha
TLRs: Toll-like receptors
TSP-1: T-cell-derived serine protease
VIP: Vasoactive intestinal peptide.

## References

[1] Neumann H (2001). Control of glial immune function by neurons. *GLIA*.

[2] Schultzberg M, Lindberg C, Aronsson AF, Hjorth E, Spulber SD, Oprica M (2007). Inflammation in the nervous system—physiological and pathophysiological aspects. *Physiology and Behavior*.

[3] Aloisi F (2001). Immune function of microglia. *GLIA*.

[4] Simard AR, Rivest S (2007). Neuroprotective effects of resident microglia following acute brain injury. *Journal of Comparative Neurology*.

[5] Wronna D (2006). Neural immune interactions: an integrative view of the directional relationship between the brain and the immune system. *Journal of Neuroimmunology*.

[6] John GR, Lee SC, Brosnan CF (2003). Cytokines: powerful regulators of glial cell activation. *The Neuroscientist*.

[7]  Chavarria A, Alcocer-Varela J (2004). Is damage in central nervous system due to inflammation. *Autoimmunity Reviews*.

[8] Lambert C, Ase AR, Séguéla P, Antel JP (2010). Distinct Migratory and cytokine responses of human microglia and macrophages to ATP. *Brain, Behavior and Immunity*.

[9] Sternberg EM (2006). Neural regulation of innate immunity: a coordinated non-specific host response to pathogens. *Nature Reviews Immunology*.

[10] Kettenmann H, Hanish UK, Noda M, Verkhratsky A (2011). Physiology of microglia. *Physiology Reviews*.

[11] Hanisch U-K, Ketenmann H (2007). Microglia: active sensor and versatile effector cells in the normal and pathological brain. *Nature Neuroscience*.

[12] Truckenmiller ME, Princiotta MF, Norbury CC, Bonnean RH (2005). Corticosterone impairs MHC class I antigen presentation by dendritic cells via reduction of peptide generation. *Journal of Neuroimmunology*.

[13] Kerschensteiner M, Meinl E, Hohlfeld R (2009). Neuro-immune crosstalk in CNS diseases. *Neuroscience*.

[14] Benn T, Halfpenny C, Sading N (2001). Glial cells as targets for cytotoxic immune mediators. *GLIA*.

[15] Goverman J (2009). Autoimmune T cells responses in the central nervous system. *Nature Review Immunology*.

[16] De Araujo EG, Da Silva GM, Dos Santos AA (2009). Neuronal cell survival. The role of interleukins. *Annals of the New York Academy of Sciences*.

[17] Pickering M, O’Connor JJ, Scharfaman HE (2007). Pro-inflammatory cytokines and their effects in the dentate gyrus. *Progress in Brain Research*.

[18] Muzio L, Martino G, Furlan R (2007). Multifaceted aspects of inflammation in multiple sclerosis: the role of microglia. *Journal of Neuroimmunology*.

[19] Kondo S, Kohsaka S, Okabe S (2011). Long-term changes of spine dynamics and microglia after transient peripheral immune response triggered by LPS in vivo. *Molecular Brain*.

[20] Czapski GA, Gajkowska B, Storsznajder JB (2010). Systemic administration of lipopolysaccharide induces molecular and morphological alterations in the hippocampus. *Brain Research*.

[21] Eggers V, Schilling A, Kox WJ, Spies C (2003). Septic encephalopathy. Diagnosis and therapy. *Anaesthesist*.

[22] Boogaard M, Ramakers BP, Alfen N (2010). Endotoxemia-induced inflammation and the effect on the human brain. *Critical Care*.

[23] Suzumura A, Takeuchi H, Zhang G, Kuno R, Mizuno T (2006). Roles of glia-derived cytokines on neuronal degeneration and regeneration. *Annals of the New York Academy of Sciences*.

[24] Jakubs K, Bonde S, Iosif RE (2008). Inflammation regulates functional integration of neurons born in adult brain. *Journal of Neuroscience*.

[25] Bloom O, Unternaehrer JJ, Jiang A (2008). Spinophilin participates in information transfer at immunological synapses. *Journal of Cell Biology*.

[26] Mateos JM, Luthi A, Savic N (2007). Synaptic modifications at CA3-CA1 synapse after chronic AMPA receptor blockade in rat hippocampal slices. *Journal of Physiology*.

[27] Shen Y, Liu SS, Zhan MY, Luo JH, Zhu JL (2010). Interleukin-2 enhances dendritic development and spinogenesis in cultured hippocampal neurons. *Anatomical Records*.

